# Smoking Status and the Five-Factor Model of Personality: Results of a Cross-Sectional Study Conducted in Poland

**DOI:** 10.3390/ijerph14020126

**Published:** 2017-01-27

**Authors:** Krzysztof Buczkowski, Małgorzata A. Basinska, Anna Ratajska, Katarzyna Lewandowska, Dorota Luszkiewicz, Alicja Sieminska

**Affiliations:** 1 Department of Family Medicine, Nicolaus Copernicus University in Torun, L. Rydygier Collegium Medicum in Bydgoszcz, M. Sklodowskiej-Curie 9, 85-094 Bydgoszcz, Poland; pieniaszek.dorota@gmail.com; 2 Institute of Psychology, Kazimierz Wielki University, Leopolda Staffa 1, 85-867 Bydgoszcz, Poland; malbasinska@wp.pl (M.A.B.); aniarat@wp.pl (A.R.); 3 Department of Palliative Care, Nicolaus Copernicus University in Torun, L. Rydygier Collegium Medicum in Bydgoszcz, Sklodowskiej-Curie 9, 85-094 Bydgoszcz, Poland; 4 Department of Oncology and Radiotherapy, Medical University of Gdansk, Dębinki 7, 80-210 Gdańsk, Poland; klewandowska@gumed.edu.pl; 5 Department of Allergology, Chair of Lung Disease, Medical University of Gdansk, Dębinki 7, 80-210 Gdańsk, Poland; asieminska@gumed.edu.pl

**Keywords:** smoking, personality traits, Five-Factor Model, health-risk behavior

## Abstract

Tobacco smoking is the single most important modifiable factor in increased morbidity and premature mortality. Numerous factors—including genetics, personality, and environment—affect the development and persistence of tobacco addiction, and knowledge regarding these factors could improve smoking cessation rates. This study compared personality traits between never, former, and current smokers, using the Five-Factor Model of Personality in a country with a turbulent smoking reduction process.*:* In this cross-sectional study, 909 Polish adults completed the Revised Neuroticism-Extraversion-Openness Personality Inventory. Our results showed that current smokers’ scores for extraversion, one of the five global dimensions of personality, were higher relative to never smokers. Neuroticism, openness to experience, agreeableness, and conscientiousness did not differ significantly according to smoking status. Facet analysis, which described each dimension in detail, showed that current smokers’ activity and excitement seeking (facets of extraversion) scores were higher relative to those of never and former smokers. In turn, current smokers’ dutifulness and deliberation (facets of conscientiousness) scores were lower than those found in former and never smokers. Never smokers scored the highest in self-consciousness (a facet of neuroticism) and compliance (a component of agreeableness). The study conducted among Polish individuals showed variation in personality traits according to their smoking status; however, this variation differed from that reported in countries in which efforts to reduce smoking had begun earlier relative to Poland. Knowledge regarding personality traits could be useful in designing smoking prevention and cessation programs tailored to individuals’ needs.

## 1. Introduction

Tobacco smoking is one of the most important factors affecting mortality rates [1,2]. It accounts for approximately 6 million deaths annually, and this number is expected to increase to 8.3 million annually by 2030 [3,4,5,6]. However, tobacco smoking is a reversible risk factor; therefore, the risk related to exposure can be reduced [7]. For this reason, it is necessary to reduce the number of people initiating smoking and increase the number of people ceasing smoking to reduce morbidity and the number of deaths attributable to smoking [8,9].

For many years, numerous countries have implemented tobacco-control policies and launched effective anti-smoking campaigns and programs aimed at reducing the consequences of smoking [7,10,11,12,13]. The results of contemporary research justify the effort made to prevent and treat nicotine addiction [14,15,16,17]. However, observations made during both large-scale, population-based programs and everyday interventions have shown that smoking cessation initiatives are not equally effective for all smokers. For instance, some smokers have made numerous unsuccessful attempts to cease smoking, which prompted us to consider individual differences in smoking behavior [18,19]. 

Smoking cessation is likely to require personal, tailored interventions that consider individual factors affecting smoking persistence [20,21]. In view of this, personality traits constitute an important factor, and increasing knowledge in this regard could enhance understanding of complex behaviors such as smoking [22,23].

Personality traits are specific, relatively stable features and the main indicators of behavior [24]. Over the last few decades, numerous studies have demonstrated the usefulness of the Five-Factor Model (FFM) of personality in describing personality traits [25,26,27]. The FFM is a hierarchical model that classifies personality traits into five factors: neuroticism, which is an inclination toward fear and low mood; extraversion, which is a tendency to be sociable and talkative and seek stimulation; openness to experience, which is characterized by creativity and intellectual, artistic, and emotional sensitivity; agreeableness, which involves trustfulness, altruism, and good-naturedness; and conscientiousness, which involves strong will, determination, responsibility, and the observance of rules. Each of these factors is represented by six specific, lower-order traits known as facets [26,28]. These traits have been shown to be partly heritable [29,30] and generalizable across cultures [27,31,32].

The beginning of research involving smokers’ personalities can be traced back to the 1970s and 1980s [33]. Initially, these studies showed that current smokers exhibited higher extraversion and lower agreeableness scores relative to those observed for former and never smokers. One of the first studies which used FFM to investigate the association between smoking and personality traits was conducted in the USA and showed that current smokers exhibited higher neuroticism and lower agreeableness and conscientiousness scores relative to those observed for never smokers [22]. However, studies conducted in Japan showed that female smokers exhibited higher extraversion and lower agreeableness scores relative to those of female nonsmokers, and male smokers exhibited higher extraversion and lower openness scores relative to those of male nonsmokers [34]. Different results were observed in a prospective research study in which assessment of personality traits began in childhood and continued until adulthood. The results showed that only low conscientiousness scores observed in childhood predicted smoking in adulthood [35]. The results of previous studies have not always been consistent, but most have shown that smokers were more neurotic and extraverted relative to nonsmokers [36,37,38]. This finding was corroborated by meta-analyses indicating that smoking was associated with high neuroticism [23,39,40] and extraversion levels [23,40] and low agreeableness [39] and conscientiousness levels [39,40]. 

Differences in personality traits were not usually large. However, considering the popularity and health risks associated with smoking, knowledge regarding even small differences could play a significant role in further reductions in numbers of smokers.

The results of state-of-the-art research indicating relationships between selected personality traits and smoking were conducted primarily in Western countries, in which the smoking reduction process began several decades ago [22,24,34,39,40,41,42]. Research involving populations in countries in which the process began more recently and took a turbulent course is missing from the literature. Although the number of smokers has decreased in all of these populations, smokers’ personality traits could differ between the two types of country. In countries in which the smoking reduction process has been established for many years, generations have grown up in an antismoking climate; however, in countries in which the smoking reduction process began later, adolescents have grown up without barriers restricting smoking and often been exposed to intensive cigarette advertising.

In addition, the difference between the two types of country could result from the fact that, with reductions in smoking rates, smokers have become less representative of overall populations, because reductions in smoking have not occurred simultaneously across all social strata.

Of the populations of post-communist countries, in which the smoking reduction process began later, that of Poland stands out because it had the highest smoking rate prior to the democratization of the political system and achieved the greatest reduction in smoking rates, catching up with many Western European countries [17,43,44].

In Poland, the sale and consumption of cigarettes increased between the Second World War and the 1980s [45], and the country had one of the highest tobacco consumption rates worldwide, at 3600 cigarettes per adult per year [46,47]. Poor knowledge regarding the consequences of smoking and a lack of anti-smoking policy led to high smoking rates in the 1980s and early 1990s, which began to decrease only when the political system changed in the 1990s [45,48]. The process of reducing cigarette consumption in Poland began much later than it did in Western countries. As in other countries, the smoking reduction process implemented in Poland involved awareness campaigns, smoking cessation interventions [49], adequate tobacco control legislation, and governmental smoking reduction programs. As a result, the number of people who ceased smoking increased, which improved the situation considerably [43,50,51]. Therefore, since improvements in socioeconomic and political situations, elimination of negative environmental factors (advertisements), and successful anti-smoking campaigns reduced smoking rates significantly, it is worth exploring individual factors that could determine smoking habits on a constant basis.

The aim of this study was to identify the FFM personality traits related to cigarette smoking in the population of a country in which the smoking reduction process was turbulent and began relatively recently.

## 2. Materials and Methods 

### 2.1. Sampling

A convenience sample of 909 participants was recruited mainly via advertisements distributed at the Institute of Psychology at Kazimierz Wielki University in Bydgoszcz, family practices cooperating with the Family Medicine Department of Ludwik Rydygier Collegium Medicum at Nicolaus Copernicus University in Torun, and the Department of Pulmonology at the Medical University of Gdansk. In addition, some participants were recruited by doctors and psychologists, who invited patients to participate in the research project voluntarily, during consultations. 

The inclusion criteria were age of 18 years or older and good communication skills. The exclusion criterion was mental disease, which could affect communication skills. All participants provided written informed consent prior to the initiation of the project. The study was conducted between March 2011 and March 2013. 

### 2.2. Measures

#### 2.2.1. Personality Assessment

Participants’ personality traits were evaluated via the Polish version of the Revised Neuroticism-Extraversion-Openness Personality Inventory (NEO-PI-R) [28,52]. The NEO-PI-R is a self-report questionnaire and has been translated into numerous languages and applied in various cultures, demonstrating its universality [53,54]. The inventory consists of 240 statements that measure five dimensions (neuroticism, extraversion, openness to experience, agreeableness, and conscientiousness), each consisting of six facets. Responses are provided using a 5-point Likert scale ranging from 1 (strongly disagree) to 5 (strongly agree).

The accuracy of the Polish version of the scale was corroborated using principal components analysis with varimax rotation [52]. The coefficients for correlations between the scales and subscales within given factors confirmed the structure of the questionnaire. Cronbach’s αs for the overall scale ranged from 0.81 to 0.86 in previous studies. Cronbach’s αs for the subscales were slightly lower; however, most ranged from 0.60 to 0.70, indicating acceptable internal consistency [52]. 

#### 2.2.2. Assessment of Smoking Behavior

Participants also completed a smoking questionnaire, which included questions concerning smoking initiation, maintenance, and cessation; reasons for smoking; and motivation for smoking cessation. Smoking status was determined according to participants’ declarations. The never-smoking group included individuals who had never smoked or reported smoking fewer than 100 cigarettes during their lifetimes [55]. The current-smoking group included individuals who had smoked at least 100 cigarettes during their lifetimes and engaged in smoking for at least 6 months [55]. The former-smoking group included individuals who had not smoked (even one puff) for at least 3 months [55]. Participants completed the questionnaires at home or the participating institutions.

### 2.3. Participants 

In total, 909 questionnaires were collected; of these, 904 were completed appropriately and included in the analysis. Participants’ demographic characteristics are shown in Table 1. Of the 904 participants, 559 (61.84%) were women aged between 20 and 84 (mean (M) = 45.98, standard deviation (SD) = 10.36) years, and 345 (38.16%) were men aged between 19 and 83 (M = 45.07, SD = 11.25) years. 

The three study groups (never smokers, former smokers, and current smokers) contained similar numbers of respondents; however, they differed significantly according to demographic characteristics. The current-smoking and never-smoking groups included significantly higher proportions of women relative to those of men (*p* = 0.007). With respect to age, participants in the former-smoking group were older relative to those in the never- and current-smoking groups, and participants in the never-smoking group were younger relative to those in the current- and former-smoking groups (*p* < 0.001). In addition, the proportion of participants in the never-smoking group who had completed secondary education was higher relative to that of those who had completed only primary education (*p* < 0.001). Most current and former smokers had completed secondary education.

### 2.4. Statistical Analyses

All statistical analyses were performed using Statistica 10 for Windows Software (StatSoft Inc., Tulsa, USA) . The assumption of homogeneity of variance was assessed using the Levene statistic. In order to characterize the investigated group, descriptive statistics of variables were used: M, SD, number (*n*), and percentage (%). In addition, chi-squared (χ^2^) tests were performed to determine the significance of differences in sex and education levels between the three groups, and a Student’s *t*-test, a Mann–Whitney U test, and an ANOVA were performed to determine the significance of differences in age, smoking onset, and the number of cigarettes smoked daily, respectively. 

In the major analysis, MANOVA was used with smoking status as the independent variable; personality factors and facets as the dependent variables; and age, sex, and education as covariates. In post hoc comparisons of never, former, and current smoker groups, Bonferroni procedure was applied.

Effect sizes were estimated using partial η^2^ values of 0.01–0.06, 0.06–0.14, and 0.14 corresponding to small, medium, and large effect sizes, respectively. Analyses were performed using T-score data.

### 2.5. Ethical Approval

The study was approved by the ethics committee for Collegium Medicum at Nicolaus Copernicus University in Torun. (Protocol registered No KB648/2010) 

## 3. Results

The mean age at which participants initiated smoking was 18.76 years (SD = 3.64; range: 10–40). Current smokers initiated smoking significantly later relative to former smokers (z = 2.16; *p* = 0.03). The mean number of cigarettes smoked by current smokers was 14.74 (SD = 9.84) per day, with a range from 0 per day for occasional smokers to 60 per day for heavy smokers. The mean numbers of cigarettes smoked by former (15.41, SD = 12.6) and current smokers did not differ significantly (z = 0.268; *p* = 0.788).

### Personality Traits

Table 2 shows the mean T-scores and SDs for personality traits for the never-, former-, and current-smoking groups. The results of the analysis of basic personality dimensions showed that extraversion differed significantly between the groups, but neuroticism, openness, agreeableness, and conscientiousness did not (F = 1.85; *p* = 0.04). Post hoc analysis showed that extraversion scores were highest in the current-smoking group (M = 50.77, SD = 9.90) and lowest in the never-smoking group (M = 48.98, SD = 10.56 *p* = 0.026). An assessment of the effect size, at the factor level, indicated that the differences between groups were small (η^2^ = 0.01).

Analyses at the facet level revealed differences between groups (F = 1.34; *p* = 0.04). Among components of extraversion, current smokers and never smokers exhibited the highest (M = 50.83, SD = 10.44) and lowest (M = 48.78, SD = 9.81) activity scores, respectively. Similarly, current smokers and never smokers showed the strongest (M = 51.47, SD = 10.21) and weakest (M = 48.87, SD = 10.15) tendencies toward excitement seeking, respectively. Post hoc tests showed significant differences in activity (*p* = 0.01) and excitement seeking (*p* < 0.02) between groups.

Analyzed groups differed significantly within self-consciousness, a facet of neuroticism. Current smokers and never smokers exhibited the lowest (M = 49.30, SD = 9.93) and highest (M = 51.23, SD = 10.43) scores, respectively. Post hoc tests showed a significant difference in self-consciousness between groups (*p* < 0.05).

Compliance, a component of agreeableness, differed significantly between groups. Former smokers and never smokers exhibited the lowest (M = 49.09, SD = 9.23) and highest (M = 51.36, SD = 10.06) compliance, respectively. Post hoc analysis showed a significant difference in compliance between groups (*p* < 0.03).

Dutifulness and deliberation, components of conscientiousness, also differed significantly between groups. Current smokers and former smokers exhibited the lowest (M = 49.02, SD = 10.03) and highest (M = 50.71, SD = 9.85) dutifulness scores, respectively. Post hoc test showed a significant difference in dutifulness between groups (*p* = 0.04). 

Similarly, current smokers and never smokers exhibited the lowest (M = 48.35, SD = 10.41) and highest (M = 51.56, SD = 9.23) deliberation scores, respectively. Post hoc estimates showed a significant difference in deliberation between groups (*p* < 0.02).

Effect sizes estimated at the facet level indicated that the differences between groups were small (η^2^ = 0.045).

## 4. Discussion

The results of the study, which was based on the FFM, demonstrated that extraversion differed significantly between current and never smokers, but the other four personality dimensions (i.e., neuroticism, openness to experience, agreeableness, and conscientiousness) did not differ significantly according to smoking status. Facet analysis, which described each dimension in detail, revealed differences in self-consciousness (a facet of neuroticism), activity and excitement seeking (facets of extraversion), compliance (a facet of agreeableness), and dutifulness and deliberation (facets of conscientiousness) between groups of never smokers, former smokers, and current smokers. 

The results also showed that, in a country in which the reduction in the smoking rate occurred rapidly within a short period, extraversion was associated with the initiation and continuation of smoking. Previous research confirmed that smokers were more extraverted, relative to nonsmokers, and this difference was most visible in populations with high rates of smoking and social acceptance of smoking [34,38]. This issue was illustrated by a meta-analysis comparing smokers from the USA and Canada with those from Spain and Japan [39]. In the USA and Canada, which had low levels of social acceptance of smoking, smokers’ and nonsmokers’ global extraversion scores did not differ significantly; conversely, in Spain and Japan, which had high levels of social acceptance of smoking when the study was conducted, smokers’ global extraversion scores were higher relative to those of nonsmokers [34,38,39]. This suggests that smokers’ extraversion levels are higher when social acceptance of smoking is greater. It is possible that greater social acceptance of smoking [56], which did not decline as rapidly as the number of smokers, in Poland was accountable for the higher levels of extraversion observed in the current study. This interpretation could be evaluated by comparing the results of previous studies conducted in the USA [57] with those of contemporary research [22]. Research conducted several decades ago reported that levels of extraversion in current smokers were significantly higher relative to those of nonsmokers [57]. However, this difference was not observed in contemporary studies. The reduction in extraversion levels in current smokers was consistent with the reduction in the number of smokers in the population and the decline in social acceptance of smoking. Therefore, when nonsmoking was a desirable social norm, respondents with high extraversion scores were more likely to decide to cease smoking and remain abstinent. 

At the facet level of extraversion, current smokers’ scores for activity and excitement seeking were high, which is a relevant finding. This neurobiological association could be explained by the stimulating effect of nicotine, which leads to increased dopaminergic activity [58], as this is considered responsible for prompting stimulation-seeking individuals to initiate and continue smoking.

Excitement seeking, in particular, has been associated with increases in the frequency of smoking. This finding has been supported by those of studies in which current smokers’ levels of excitement seeking were higher relative to those observed in nonsmokers, even though no differences in global extraversion were observed between the two groups [22].

It is likely that extraverts are more prone to environmental factors affecting smoking and change their smoking attitudes (regarding smoking initiation or cessation) in accordance with the level and duration of smoking acceptance or the anti-smoking climate in a given country. Therefore, influencing this dimension of current smokers’ personalities could contribute to further reductions in smoking.

Although no significant differences in global conscientiousness were observed between groups, the results showed that current smokers’ and never smokers’ deliberation scores were lowest and highest, respectively. This could indicate that never smokers thought about their actions carefully and considered the consequences, while current smokers acted rashly without deep reflection. Dutifulness scores, which were lowest among current smokers and highest among former smokers, may indicate the usefulness of this facet in smoking cessation. Our results are partly consistent with those of the study conducted by Terracciano et al. [22]. One of the first studies examining smokers via the NEO-PI-R included middle-aged and elderly populations, and current smokers’ scores for all facets of conscientiousness were lower relative to those observed for never smokers. Similar results were observed in later studies involving drug users [59], in which low conscientiousness levels were related to addiction to not only nicotine but also other substances such as cocaine and heroin. Results of research examining risky health behavior showed that of all of the personality traits, low conscientiousness played the most important role in such behavior [60]. Kubicka et al. [35] reported another interesting observation concerning conscientiousness in a prospective study involving 24-year follow up, in which low conscientiousness levels in childhood predicted smoking in adulthood, but extraversion and neuroticism levels did not.

No significant differences between studied groups were found in the global neuroticism in the current study, while, among facets of this factor, the single negative correlation was found between self-consciousness and smoking. These results differ from those of many previous studies, such as those conducted by Terracciano [22,59], and meta-analyses, such as those conducted by Munafo et al. [23], Malouff et al. [39], and Hakulinen [40], in which high neuroticism scores were associated with current smoking, with only a few studies (e.g., Yoshimura [34]) reporting incongruous findings. As high neuroticism levels were observed particularly frequently in current smokers, researchers posited that it could both facilitate smoking initiation and hinder smoking cessation [22,37,39,61,62,63]. One explanation for these findings could be that individuals resorted to cigarette smoking as a means of self-medication, to reduce tension or anxiety [64]. In addition, the impact of nicotine on smokers’ well-being has been demonstrated in studies examining the biological effects of smoking, which occurred mainly via its influence on nicotine receptors in the brain’s reward system [65,66]. 

Cultural, economic, and political determinants of smoking and overall levels of neuroticism in the study population should be considered in the interpretation of differences in research outcomes and identification of reasons why neuroticism did not differ significantly between the never-, former-, and current-smoking groups in the current study. With respect to the economic situation, it should be noted that most studies demonstrating an association between smoking and neuroticism were conducted in countries that were wealthier and more developed, relative to Poland [67]. For many years, Poland was a communist country with a low gross domestic product (GDP) a high smoking rate, and low social awareness of the risks of smoking, and the country’s smoking rate continued to increase until democratization, which began 25 years ago [44]. Bosnia provides another example of the effects of a country’s political situation on smoking prevalence. The Bosnian conflict between 1992 and 1995 was hypothesized to be a direct cause of high smoking rates in family physicians and nurses in Bosnia and Herzegovina, which decreased significantly subsequent to the war [68].

Another perspective on the interpretation of the results focuses on differences in personality traits between inhabitants of different countries. Previous research examining personality differences between individuals from different cultures and countries showed that neuroticism levels in inhabitants of Central and Eastern European countries were higher relative to those observed in inhabitants of Western countries, regardless of smoking status [69,70]. The high levels of neuroticism observed in the overall study population could explain the lack of differences between the three groups in the current study.

Moreover, participants’ age could also have affected the results. The average age of the participants in the current study was lower relative to those of participants in the studies demonstrating a relationship between neuroticism and smoking [22,59]. Sampling could also have exerted an impact on the findings, as the participants were recruited from a general population but were not representative of the overall Polish population. In addition, the number of women in the sample was higher relative to that of men, and the existence of psychological and biological differences in smoking between men and women is well known [71].

When comparing the results of the current study with those of previous studies, it is necessary to note the time span between them. More than one or two decades have passed since many of the previous studies analyzing the correlation between personality and smoking were conducted [22,23,34,35], and changes in many external factors affecting smoking initiation, maintenance, and cessation have occurred during recent years. The last few years have also seen the implementation of smoking restrictions, a ban on cigarette advertising, a considerable increase in the cost of cigarettes, and increasing numbers of smoking cessation initiatives [13,72]. These factors provide strong encouragement for smoking cessation and motivate smokers to cease smoking [73]. 

### Strengths and Limitations

The main strength of this study was that it included an underexamined population; research from the USA and Western Europe is most prevalent in the literature, and studies conducted in less well-developed countries is relatively scarce. Despite this, the study was subject to some limitations, which should be considered when interpreting the research findings. First, the sample was not representative of the overall population. While the sample consisted of people of different ages with various educational levels, women were overrepresented in the study. Second, participants’ smoking status was self-reported, while the use of objective tests, such as measurement of carbon monoxide or cotinine levels, is a preferable method of confirming information regarding abstinence; however, previous research has shown that self-reported data regarding smoking is generally reliable [74]. Similarly, no psychometric instruments were administered to screen for depression or anxiety, and data regarding potential mental disorders were self-reported. 

## 5. Conclusions 

This study confirmed the associations between current smoking and high extraversion and its facets (activity and excitement seeking) in the population of a country in which the implementation of the smoking reduction process was both turbulent and relatively recent. Furthermore, the results revealed an association between high levels of deliberation, which is a facet of conscientiousness, and never-smoking. The findings could be used in the design of new smoking prevention programs and tailored anti-smoking interventions. Accordingly, the consideration of conscientiousness, particularly the deliberation facet, could be useful in the development of interventions to prevent smoking initiation, and knowledge of the association between extraversion and smoking could be used in the establishment of adequate anti-smoking policy intended to reduce social acceptance of smoking, which should encourage extraverted smokers to cease smoking. This could be particularly effective in countries in which the proportion of extraverted smokers is larger relative to that of extraverted nonsmokers. In future studies, it would be useful to determine whether the effectiveness of different types of psycho- and pharmacotherapy are related to smokers’ personality traits, and tailoring treatment and consultations to the smokers’ personality profiles is worthwhile.

## Figures and Tables

**Table 1:** Demographic characteristics of 904 participants examined in the study according to smoking status.

	Never Smokers (*n* = 294)	Former Smokers (*n* = 277)	Current Smokers (*n* = 333)
Gender *			
Female (%)	192 (65.3)	150 (54.1)	217 (65.2)
Male (%)	102 (34.7)	127 (45.9)	116 (34.8)
Age: in years (M ± SD) *	43.5 ± 11.18	48.1 ± 10.34	45.4 ± 10.19
Age range	19–84	22–69	20–75
Education *			
Basic and Vocational (%)	28 (9.6)	56 (20.2)	74 (22.2)
Secondary (%)	101 (34.4)	111 (40)	146 (43.8)
Higher (%)	164 (56)	110 (39.8)	113 (33.9)

M: mean; SD: Standard deviation. * *p* < 0.05.

**Table 2:** Personality traits presented as mean T-scores and standard deviations for never, former and current smokers.

NEO-PI-R Scales	Never Smokers *n* = 294		Former Smokers *n* = 277		Current Smokers *n* = 333		
	M	SD	M	SD	M	SD	*p*
Neuroticism	50.40	10.31	49.51	9.58	50.05	10.06	ns
Extraversion	48.98	10.56	50.14	9.41	50.77	9.90	0.026 ^a^
Openness	50.77	9.88	49.47	10.33	49.75	9.77	ns
Agreeableness	50.36	10.57	49.77	9.23	49.87	10.10	ns
Conscientiousness	50.55	9.58	50.52	10.19	49.08	10.15	ns
N1: Anxiety	50.41	9.82	49.68	9.75	49.90	10.35	ns
N2: Angry Hostility	49.80	10.38	49.58	9.85	50.51	9.76	ns
N3: Depression	50.44	10.36	49.76	9.77	49.80	9.85	ns
N4: Self-consciousness	51.23	10.43	49.50	9.50	49.30	9.93	<0.05 ^a,b^
N5: Impulsiveness	49.64	9.73	49.94	10.04	50.35	10.19	ns
N6: Vulnerability	50.49	10.25	49.38	9.67	50.07	10.02	ns
E1: Warmth	50.30	10.51	50.05	9.15	49.68	10.21	ns
E2: Gregariousness	49.78	10.02	49.56	9.66	50.55	10.24	ns
E3: Assertiveness	49.37	10.76	50.48	9.40	50.14	9.75	ns
E4: Activity	48.78	9.81	50.30	9.53	50.83	10.44	0.01 ^a^
E5: Excitement-Seeking	48.87	10.15	49.44	9.37	51.47	10.21	<0.02 ^a,c^
E6: Positive Emotions	49.73	10.26	49.56	9.68	50.60	10.00	ns
O1: Fantasy	50.50	10.56	49.61	9.69	49.87	9.72	ns
O2: Aesthetics	50.76	9.53	50.01	9.94	49.30	10.40	ns
O3: Feelings	50.44	9.95	49.98	10.31	49.62	9.76	ns
O4: Actions	49.80	10.07	49.68	9.99	50.43	9.92	ns
O5: Ideas	50.80	10.13	49.49	9.51	49.71	10.24	ns
O6: Values	50.08	9.82	49.33	10.12	50.48	10.03	ns
A1: Trust	49.98	9.98	50.27	9.21	49.78	10.63	ns
A2: Straightforwardness	50.46	9.81	50.28	9.43	49.35	10.58	ns
A3: Altruism	50.35	10.50	50.02	9.18	49.66	10.19	ns
A4: Compliance	51.36	10.06	49.09	9.23	49.55	10.44	<0.03 ^a,b^
A5: Modesty	49.27	10.46	50.18	9.47	50.49	9.98	ns
A6: Tender-mindedness	49.66	10.18	50.14	9.36	50.17	10.34	ns
C1: Competence	50.09	9.93	50.52	9.81	49.48	10.19	ns
C2: Order	50.42	9.77	50.47	9.67	49.23	10.41	ns
C3: Dutifulness	50.43	10.03	50.71	9.85	49.02	10.03	0.04 ^c^
C4: Achievement Striving	50.16	10.00	50.02	10.33	49.83	9.71	ns
C5: Self-Discipline	49.44	9.92	50.40	10.31	50.14	9.78	ns
C6: Deliberation	51.56	9.23	50.33	9.99	48.35	10.41	<0.02 ^a^

Note: Statistically significant differences after Bonferroni correction: ^a^ Statistically significant differences between never smokers and current smokers; ^b^ Statistically significant differences between never smokers and former smokers; ^c^ Statistically significant differences between former smokers and current smokers; ns: Statistically not significant differences. NEO-PI-R: Revised Neuroticism-Extraversion-Openness Personality Inventory. N1–N6: facets of Neuroticism; E1–E6: facets of Extraversion; O1-O6: facets of Openness to experience; A1–A6: facets of Agreeableness; C1–C6: facets of Conscientiousness.
